# Pity the Poor Panel Patient

**Published:** 1923-08

**Authors:** R. W. Harris


### PITY THE POOR PANEL PATIENT!

By R. W. HARRIS.

"PANEL patients are bought and sold like so many pounds of tea." It is Mr. Lovat Fraser who speaks. It might, perhaps, be thought that, as Mr. Fraser was anxious to "get rid of the panels," in the columns of the *Sunday Pictorial*, he was merely seeking to be picturesque. Therein you do him wrong, for Mr. Fraser has been writing a serious article, and various doctors who have written him very silly letters in which they bawl out "stunt"—it is, again, Mr. Lovat Fraser who speaks—have been rebuked, and informed that the article "was written after consultation with eminent medical men, and with much documentary evidence, including all the debates in Parliament, before me."

### Dr. Cox Answers Mr. Fraser.

Dr. Cox has been admitted to the columns of the *Sunday Pictorial* to reply to Mr. Fraser as follows: "Panel patients are bought and sold just like private patients. When a doctor retires or dies, he or his executors sell the introduction to his patients—all his patients—to a successor, who takes his chance as to whether the patients will come to him or not. Both panel and private patients can please themselves." Whereupon Mr. Lovat Fraser replies: "It is not disputed that panel patients are bought and sold, and there is 'nothing in this point.'" If by this we are to understand that there was nothing, after all, in the pound-of-tea idea, well and good, but in that case it is a little difficult to know how so serious a writer came to mislead those who hang upon his well-weighed words. It must, however, we fear, be inferred that Mr. Fraser fails to see any point in Dr. Cox's reply.

### Panel Patients as Goodwill.

We are, therefore, left to pity the poor panel patient who is being passed over the counter like the dumb, unresisting pound of tea. But if Dr. Cox is right in saying that what takes place is just what happens in private practice, how is the evil going to be remedied by getting rid of the panels? A panel is merely a list of the patients for whom a doctor has accepted responsibility, and if he dies or retires from practice, a prospective purchaser is told by the doctor or his executors as the case may be: "Here is a list of the private patients forming part of this practice, and here is another list, in which are set forth the names of the panel patients, forming the other part of the practice. The panel patients, just like the private patients, have chosen the doctor who has been practising at this address to be their doctor, and a good many of them will doubtless continue to come to this address in the future. There is therefore what is called a goodwill value to the practice which it is now desired to sell." Those critics of the panel system who desire to substitute a salaried medical service may quite logically find in this method of transferring practices from one doctor to another something which is not at all satisfactory in their view. Nor is it to be expected that in the event of a change of system at any time the Government would recognise that a doctor has any sort of vested interest in his panel patients. But how those who advocate a return, or at any rate a closer approximation, to the conditions of private practice can object to a doctor selling the goodwill of his practice is a question to which, frankly, I am puzzled to find an answer.

### The Panel Patient's Freedom.

When a practice changes hands, not only has every patient on the outgoing doctor's list a right to choose any other doctor in the area, but he is definitely told so, and also reminded of the fact after a suitable interval. He is, in fact, left free to choose his own doctor for a long period—anything up to eighteen months—before the necessary action is taken, if he has not then chosen a doctor, to place him on some doctor's list. Even if this is done, he will be free to change over to some other doctor of his choice. Under the Regulations as they at present stand this can only be done at certain half-yearly periods unless there is a mutual arrangement. But the doctors themselves are asking for an alteration in their terms of service under which the insured person would have the right to change his doctor at any time. This unrestricted right of transfer at any time may not prove an unmixed blessing, so far as Insurance funds are concerned, but it would at least be a complete answer to those who say that a patient is unfairly bound hand and foot to a panel doctor to be able to reply that he is free to go to another doctor at any time.

### Lo! The Poor Patient.

Although, therefore, it may be said that patients may be bought and sold, it is obvious to the meanest intelligence that the whole point of Mr. Lovat Fraser's statement was that there was something shocking to the public conscience in the thought that the poor unsuspecting panel patients were being trafficked between one doctor and another without having any say in the matter. Indeed, on turning to a cutting from another newspaper I find the same idea developed in headlines as follows:—

"Panel Patient Scandal.

Doctors Who 'Sell' Clients

So Much a Head."

Under these headlines I read of dreadful things which are supposed to be going on, winding up with the observation that what the patients will think about the whole business remains to be seen. My own feeling is that when these poor misguided people really learn the facts, they will think that someone has been pulling the writer's leg. As to Mr. Lovat Fraser, I refuse to think that after Dr. Cox's statement he is any longer under any misapprehension in the matter; but he was certainly misinformed in the first instance, with the result that he rather gave himself away with that pound of tea.

